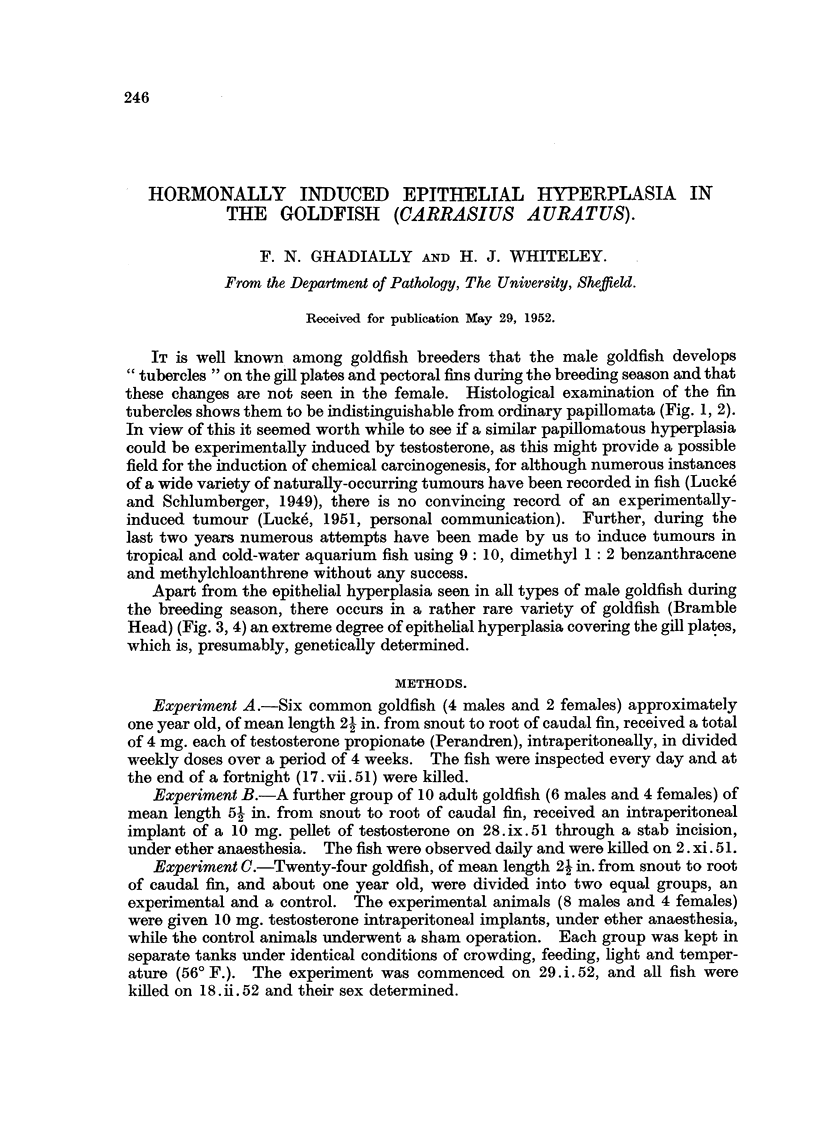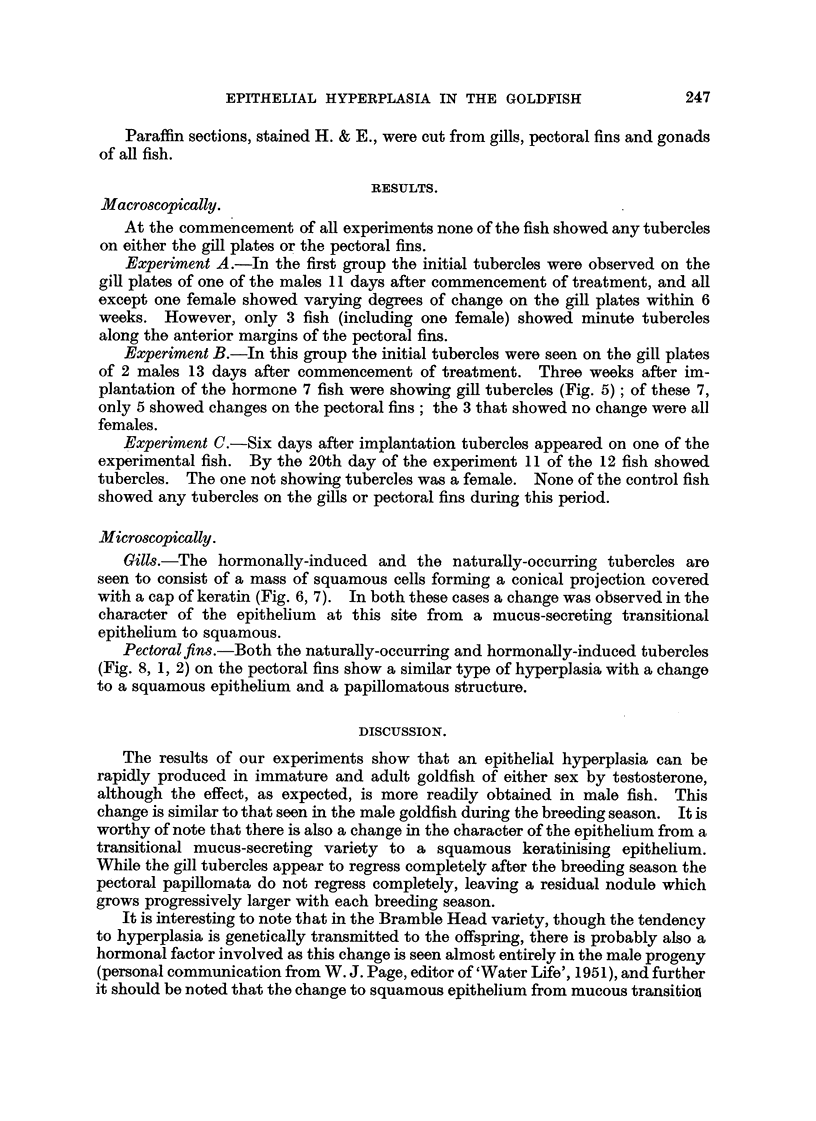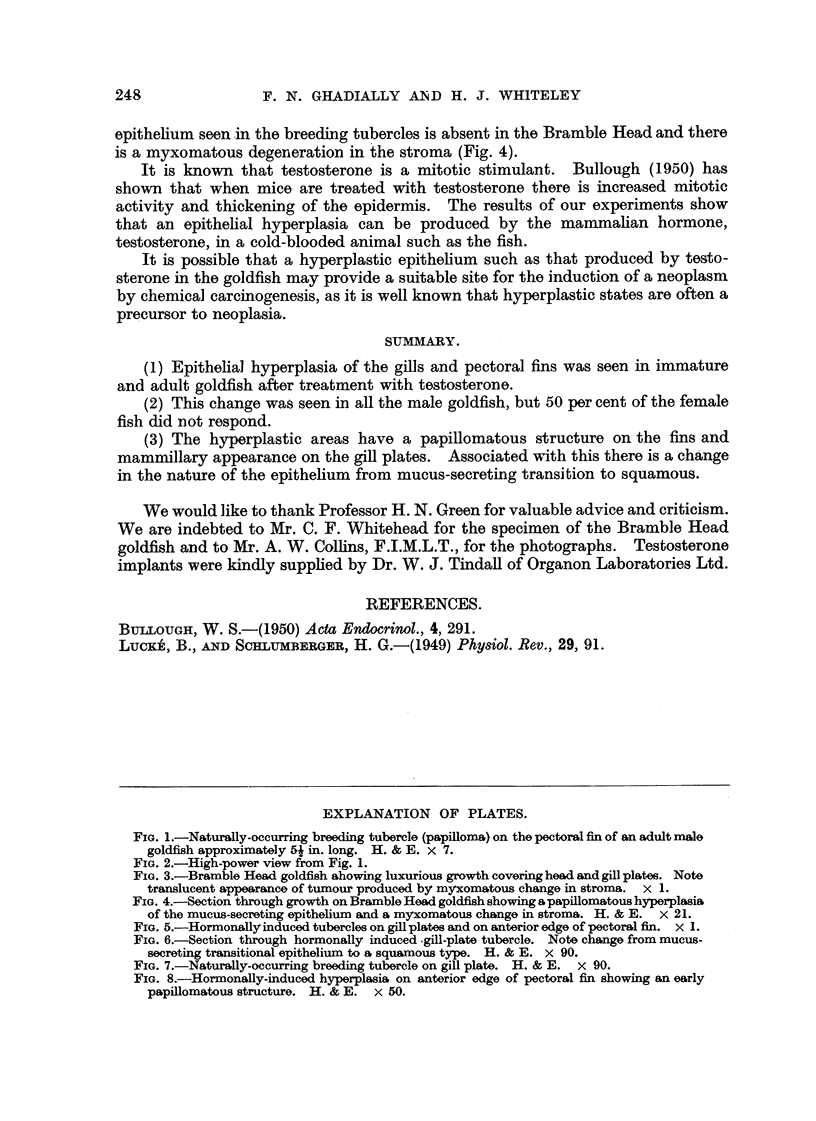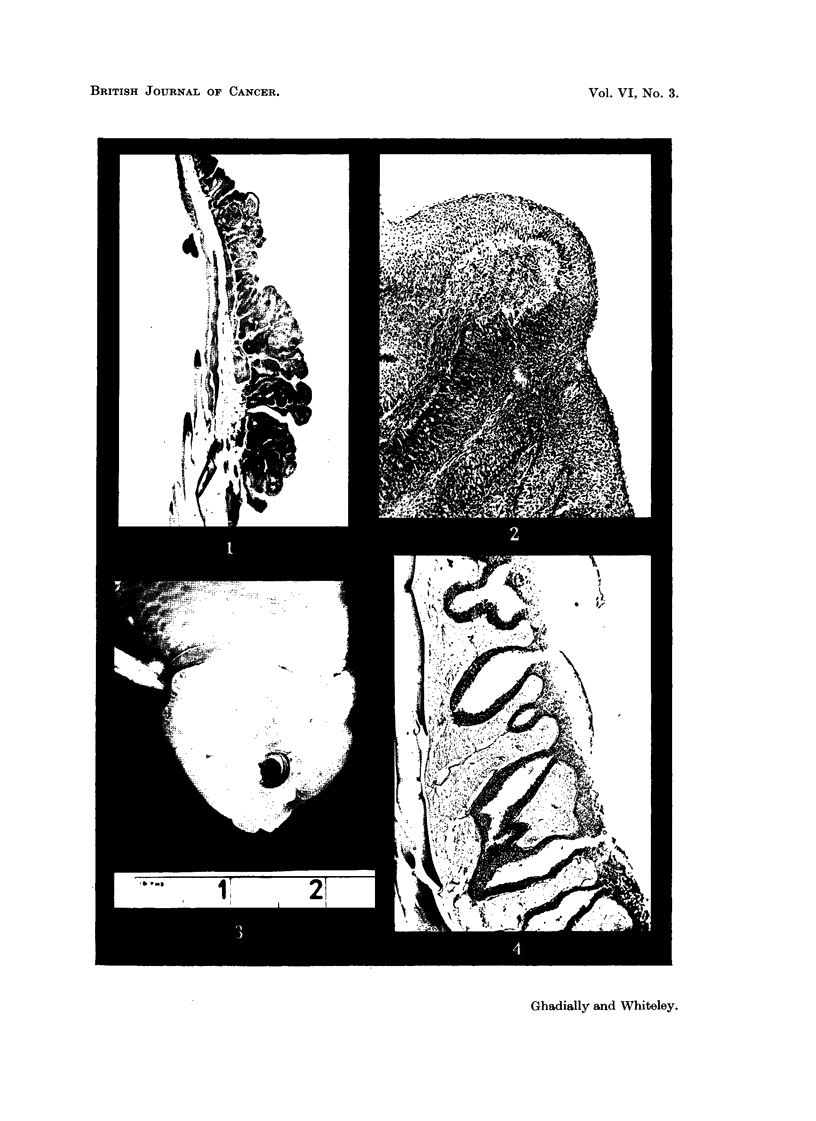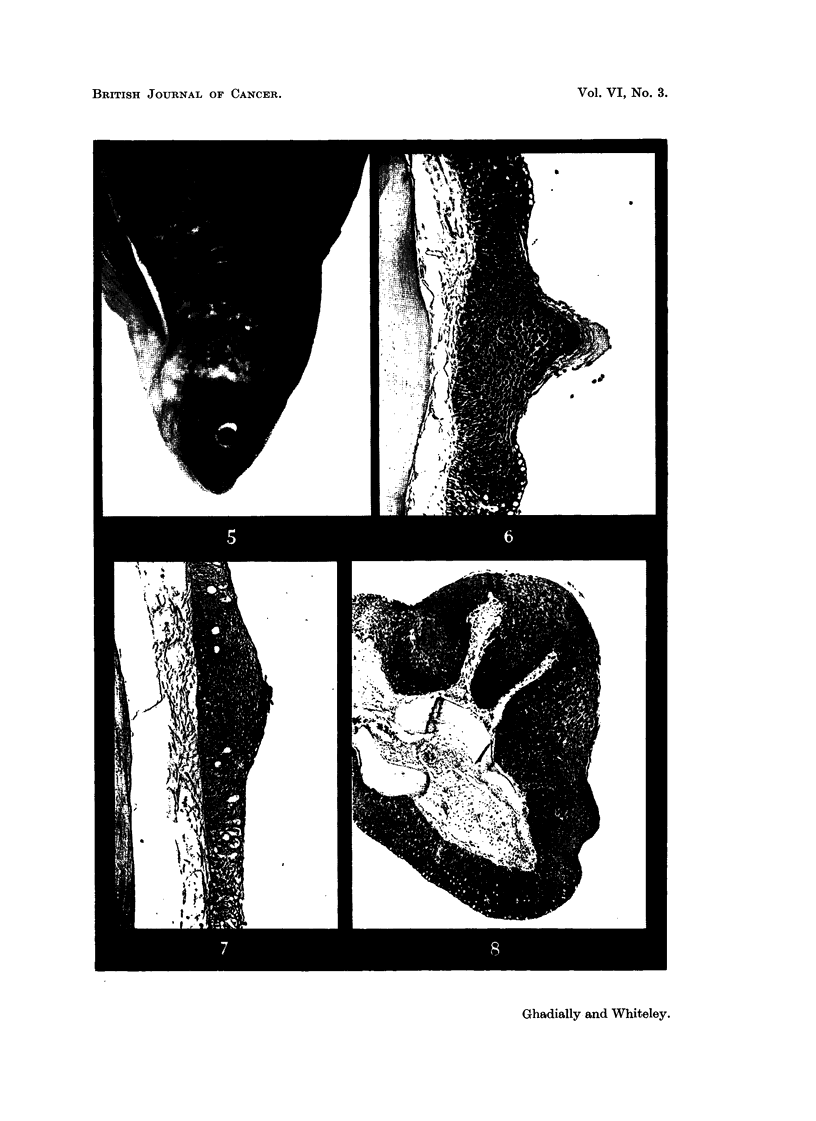# Hormonally Induced Epithelial Hyperplasia in the Goldfish (Carrasius auratus)

**DOI:** 10.1038/bjc.1952.28

**Published:** 1952-09

**Authors:** F. N. Ghadially, H. J. Whiteley

## Abstract

**Images:**


### HORMONALLY INDUCED EPITHELIAL HYPERPLASIA IN THE GOLDFISH (*CARRASIUS AURATUS*).

F. N. GHADIALLY AND H. J. WHITELEY.

*From the Department of Pathology, The University, Sheffield.*

Received for publication May 29, 1952.

IT is well known among goldfish breeders that the male goldfish develops " tubercles " on the gill plates and pectoral fins during the breeding season and that these changes are not seen in the female. Histological examination of the fin tubercles shows them to be indistinguishable from ordinary papillomata (Fig. 1, 2). In view of this it seemed worth while to see if a similar papillomatous hyperplasia could be experimentally induced by testosterone, as this might provide a possible field for the induction of chemical carcinogenesis, for although numerous instances of a wide variety of naturally-occurring tumours have been recorded in fish (Lucké and Schlumberger, 1949), there is no convincing record of an experimentally-induced tumour (Lucké, 1951, personal communication). Further, during the last two years numerous attempts have been made by us to induce tumours in tropical and cold-water aquarium fish using 9 : 10, dimethyl 1 : 2 benzanthracene and methylchloanthrene without any success.

Apart from the epithelial hyperplasia seen in all types of male goldfish during the breeding season, there occurs in a rather rare variety of goldfish (Bramble Head) (Fig. 3, 4) an extreme degree of epithelial hyperplasia covering the gill plates, which is, presumably, genetically determined.

### METHODS.

*Experiment A.*—Six common goldfish (4 males and 2 females) approximately one year old, of mean length $2\frac{1}{2}$ in. from snout to root of caudal fin, received a total of 4 mg. each of testosterone propionate (Perandren), intraperitoneally, in divided weekly doses over a period of 4 weeks. The fish were inspected every day and at the end of a fortnight (17.vii.51) were killed.

*Experiment B.*—A further group of 10 adult goldfish (6 males and 4 females) of mean length $5\frac{1}{2}$ in. from snout to root of caudal fin, received an intraperitoneal implant of a 10 mg. pellet of testosterone on 28.ix.51 through a stab incision, under ether anaesthesia. The fish were observed daily and were killed on 2.xi.51.

*Experiment C.*—Twenty-four goldfish, of mean length $2\frac{1}{2}$ in. from snout to root of caudal fin, and about one year old, were divided into two equal groups, an experimental and a control. The experimental animals (8 males and 4 females) were given 10 mg. testosterone intraperitoneal implants, under ether anaesthesia, while the control animals underwent a sham operation. Each group was kept in separate tanks under identical conditions of crowding, feeding, light and temperature (56° F.). The experiment was commenced on 29.i.52, and all fish were killed on 18.ii.52 and their sex determined.

Paraffin sections, stained H. & E., were cut from gills, pectoral fins and gonads of all fish.

### RESULTS.

*Macroscopically.*

At the commencement of all experiments none of the fish showed any tubercles on either the gill plates or the pectoral fins.

*Experiment A.*—In the first group the initial tubercles were observed on the gill plates of one of the males 11 days after commencement of treatment, and all except one female showed varying degrees of change on the gill plates within 6 weeks. However, only 3 fish (including one female) showed minute tubercles along the anterior margins of the pectoral fins.

*Experiment B.*—In this group the initial tubercles were seen on the gill plates of 2 males 13 days after commencement of treatment. Three weeks after implantation of the hormone 7 fish were showing gill tubercles (Fig. 5) ; of these 7, only 5 showed changes on the pectoral fins ; the 3 that showed no change were all females.

*Experiment C.*—Six days after implantation tubercles appeared on one of the experimental fish. By the 20th day of the experiment 11 of the 12 fish showed tubercles. The one not showing tubercles was a female. None of the control fish showed any tubercles on the gills or pectoral fins during this period.

*Microscopically.*

*Gills.*—The hormonally-induced and the naturally-occurring tubercles are seen to consist of a mass of squamous cells forming a conical projection covered with a cap of keratin (Fig. 6, 7). In both these cases a change was observed in the character of the epithelium at this site from a mucus-secreting transitional epithelium to squamous.

*Pectoral fins.*—Both the naturally-occurring and hormonally-induced tubercles (Fig. 8, 1, 2) on the pectoral fins show a similar type of hyperplasia with a change to a squamous epithelium and a papillomatous structure.

### DISCUSSION.

The results of our experiments show that an epithelial hyperplasia can be rapidly produced in immature and adult goldfish of either sex by testosterone, although the effect, as expected, is more readily obtained in male fish. This change is similar to that seen in the male goldfish during the breeding season. It is worthy of note that there is also a change in the character of the epithelium from a transitional mucus-secreting variety to a squamous keratinising epithelium. While the gill tubercles appear to regress completely after the breeding season the pectoral papillomata do not regress completely, leaving a residual nodule which grows progressively larger with each breeding season.

It is interesting to note that in the Bramble Head variety, though the tendency to hyperplasia is genetically transmitted to the offspring, there is probably also a hormonal factor involved as this change is seen almost entirely in the male progeny (personal communication from W. J. Page, editor of 'Water Life', 1951), and further it should be noted that the change to squamous epithelium from mucous transition epithelium seen in the breeding tubercles is absent in the Bramble Head and there is a myxomatous degeneration in the stroma (Fig. 4).

It is known that testosterone is a mitotic stimulant. Bullough (1950) has shown that when mice are treated with testosterone there is increased mitotic activity and thickening of the epidermis. The results of our experiments show that an epithelial hyperplasia can be produced by the mammalian hormone, testosterone, in a cold-blooded animal such as the fish.

It is possible that a hyperplastic epithelium such as that produced by testosterone in the goldfish may provide a suitable site for the induction of a neoplasm by chemical carcinogenesis, as it is well known that hyperplastic states are often a precursor to neoplasia.

### SUMMARY.

(1) Epithelial hyperplasia of the gills and pectoral fins was seen in immature and adult goldfish after treatment with testosterone.

(2) This change was seen in all the male goldfish, but 50 per cent of the female fish did not respond.

(3) The hyperplastic areas have a papillomatous structure on the fins and mammillary appearance on the gill plates. Associated with this there is a change in the nature of the epithelium from mucus-secreting transition to squamous.

We would like to thank Professor H. N. Green for valuable advice and criticism. We are indebted to Mr. C. F. Whitehead for the specimen of the Bramble Head goldfish and to Mr. A. W. Collins, F.I.M.L.T., for the photographs. Testosterone implants were kindly supplied by Dr. W. J. Tindall of Organon Laboratories Ltd.

### REFERENCES.

BULLOUGH, W. S.—(1950) *Acta Endocrinol.*, **4**, 291.
LUCKÉ, B., AND SCHLUMBERGER, H. G.—(1949) *Physiol. Rev.*, **29**, 91.

---

### EXPLANATION OF PLATES.

FIG. 1.—Naturally-occurring breeding tubercle (papilloma) on the pectoral fin of an adult male goldfish approximately 5½ in. long. H. & E. × 7.
FIG. 2.—High-power view from Fig. 1.
FIG. 3.—Bramble Head goldfish showing luxurious growth covering head and gill plates. Note translucent appearance of tumour produced by myxomatous change in stroma. × 1.
FIG. 4.—Section through growth on Bramble Head goldfish showing a papillomatous hyperplasia of the mucus-secreting epithelium and a myxomatous change in stroma. H. & E. × 21.
FIG. 5.—Hormonally induced tubercles on gill plates and on anterior edge of pectoral fin. × 1.
FIG. 6.—Section through hormonally induced gill-plate tubercle. Note change from mucus-secreting transitional epithelium to a squamous type. H. & E. × 90.
FIG. 7.—Naturally-occurring breeding tubercle on gill plate. H. & E. × 90.
FIG. 8.—Hormonally-induced hyperplasia on anterior edge of pectoral fin showing an early papillomatous structure. H. & E. × 50.